# Autonomic dysfunction, diabetes and metabolic syndrome

**DOI:** 10.1111/jdi.13691

**Published:** 2021-10-27

**Authors:** Tae Yang Yu, Moon‐Kyu Lee

**Affiliations:** ^1^ Division of Endocrinology and Metabolism Department of Medicine Wonkwang Medical Center Wonkwang University School of Medicine Iksan Korea; ^2^ Division of Endocrinology and Metabolism Department of Internal Medicine Uijeongbu Eulji Medical Center Eulji University School of Medicine Uijeongbu Korea

## Abstract

Relationships among autonomic nervous system, diabetes and metabolic syndrome.

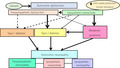

Autonomic neuropathy is most often associated with advanced diabetes mellitus, either type 1 or type 2. The chronic diabetes complication can occur in various forms, and affects the cardiovascular, gastrointestinal, endocrine and genitourinary systems.

One of the severe complications frequently under‐recognized is cardiovascular autonomic neuropathy (CAN). The CAN Subcommittee of the Toronto Consensus Panel on Diabetic Neuropathy defined CAN as, “impairment of cardiovascular autonomic control in patients with established diabetes after excluding other causes.”^1^. This CAN is the major cause of cardiovascular morbidity and overall mortality, and clinical course varies with resting tachycardia, orthostatic hypotension, exercise intolerance and silent myocardial ischemia. The affliction can present without any symptoms, especially at the early stage, and often is diagnosed at an advanced stage. Early detection of CAN can allow disease reversal, indicating the importance of early detection.

CAN also can occur in metabolic syndrome (MetS) or precede the development of diabetes mellitus and MetS. In this editorial, we will briefly review the latest evidence regarding the relationships of CAN with type 2 diabetes and MetS.

## PATHOPHYSIOLOGY OF CAN

The pathophysiology of CAN is multifactorial, and certain parts of it are unclear. However, hyperglycemia is considered the most important factor in the pathogenesis. A hyperglycemic condition can induce neuronal injury through oxidative stress, polyol pathway alteration, protein kinase C activation and advanced glycation end‐products formation, which also can be activated in non‐hyperglycemic metabolic stress conditions, such as prediabetes and metabolic syndrome.

Damage to the parasympathetic nervous system is followed by sympathetic nervous system injury, usually first affecting the vagus nerve, the longest parasympathetic nerve. As a result, the relative sympathetic tone increases, causing resting tachycardia; in the advanced stage, the sympathetic nerve is also affected. Sympathetic nerve injury could cause exercise intolerance and orthostatic hypotension. Furthermore, chest pain could be missed due to sensory nerve injuries in the heart, which can lead to silent myocardial ischemia.

## DIAGNOSIS OF CAN

A diagnostic approach to CAN should be based on assessment of both sympathetic and parasympathetic functions. There are several methods to diagnose CAN; those proposed by Ewing and Clarke^2^ are widely used, and mainly involve measuring changes in heart rate and blood pressure with maneuvers. Diagnostic assessment of CAN could be carried out by cardiac autonomic reflex tests based on certain criteria (Table 1), and is considered the gold standard in autonomic testing, as stated by the Toronto Consensus^1^. Cardiac autonomic reflex tests are useful in clinical practice, because they are non‐invasive and reproducible, and have objective parameters. Indicators of parasympathetic function include heart rate response to deep breathing, heart rate response to standing and heart rate response to the Valsalva maneuver. The evaluation of sympathetic function includes blood pressure response to standing and blood pressure response to sustained handgrip.

**Table 1:** Battery of cardiovascular reflex tests

Tests	Technique	Normal response	Borderline value	Abnormal value
Tests to investigate parasympathetic function				
Heart rate response to deep breathing	Beat‐to‐beat variation while the patient breathes in and out (b.p.m.)	≥15	11–14	≤10
Heart rate response to standing	30:15 ratio (ratio of R‐R interval measured at beats 30 and 15 after standing)	≥1.04	1.01–1.03	≤1.00
Heart rate response to Valsalva maneuver	Valsalva ratio (ratio of longest to shortest R‐R interval measured while performing the Valsalva maneuver)	≥1.21	1.11–1.20	≤1.10
Tests to investigate sympathetic function				
Blood pressure response to standing	Decrease in systolic pressure by supine to standing	≤10 mmHg	11–29 mmHg	≥30 mmHg
Blood pressure response to sustained handgrip	Increase in diastolic pressure by squeezing a handgrip dynamometer	≥16 mmHg	11–15 mmHg	≤10 mmHg

Early CAN could be suspected if any one of the aforementioned five tests is abnormal; if two or more tests show abnormality, definitive CAN can be diagnosed. In addition, accompanying orthostatic hypotension indicates severe or advanced CAN^1^.

In addition to these parameters, heart rate recovery (HRR) and heart rate variability (HRV) are used for cardiac autonomic function tests. HRR after exercise is a tool to assess cardiac autonomic function, and is calculated as the difference between the peak heart rate during exercise and the heart rate at a specific time interval after recovery^3^. This is calculated as the peak heart rate minus the resting heart rate after a 1‐min rest (HRR1), 2‐min rest (HRR2) and 3‐min rest (HRR3).

HRV is a non‐invasive marker of autonomic imbalance and vagal activity. Increased sympathetic tone decreased HRV, whereas increased parasympathetic tone increased HRV. Although the gold standard protocol for HRV estimation is 24‐h measurement, short‐term HRV recordings are used widely. They appear to be the most commonly studied source of HRV examination, potentially because of the associated ease of recording. HRV is analyzed in both time and frequency domains according to the standards recommended by the Task Force of the European Society of Cardiology and the North American Society of Pacing and Electrophysiology^4^. Time domain measurements include the standard deviation of the normal‐to‐normal interval (ms) and the root mean square differences of successive normal‐to‐normal intervals (ms). The standard deviation of the normal‐to‐normal interval is a marker of overall autonomic modulation, whereas the root mean square differences of successive normal‐to‐normal intervals reflects the cardiac parasympathetic drive. Frequency domain measurements include total (0–0.4 Hz; ms^2^), low‐frequency (LF; 0.04–0.15 Hz; ms^2^) and high‐frequency (HF; 0.15–0.4 Hz; ms^2^) powers, and the LF/HF ratio. LF and HF norms in percentile units reflect changes in sympathetic and parasympathetic regulation, and the LF/HF ratio is a measure of sympathovagal balance.

## RELATIONSHIP BETWEEN CAN AND DEVELOPMENT OF TYPE 2 DIABETES

In addition to being a complication of diabetes, autonomic dysfunction can precede the development of type 2 diabetes. As the autonomic nervous system plays a critical role in HRR after exercise, delayed HRR could represent autonomic dysfunction. Indeed, delayed HRR after a graded exercise treadmill test was reported to be an independent predictor of incident type 2 diabetes.

It recently has been shown that delayed HRR is a potent predictor for the development of type 2 diabetes in men, even after adjustments for confounding parameters, such as hemoglobin A1c, fasting plasma glucose, homeostasis model assessment of insulin resistance and homeostasis model assessment of β‐cell function in a retrospective longitudinal cohort study^5^. Interestingly, the time point for HRR after exercise to predict the development of type 2 diabetes was 1 min after exercise; that is, HRR1 was the most potent predictor of incident type 2 diabetes, whereas HRR2 and HRR3 did not show statistical significance.

This indicates that early dysfunction of the parasympathetic nervous system plays a role in the development of type 2 diabetes through effects on the pancreas, which is innervated by parasympathetic fibers to stimulate insulin secretion. In addition, insulin resistance and associated hyperinsulinemia can lead to parasympathetic fiber damage and a decrease in HRR. Inclusion of HRR after exercise in established cardiovascular reflex tests is recommended. The exact mechanism(s) contributing to the delayed HRR values after exercise to predict the development of type 2 diabetes warrants further studies.

In addition, it was shown that decreased HRV might precede the development of cardiovascular disease or diabetes. Recently, it has been shown in a prospective cohort study that abnormal HRV, especially decreased vagal activity and deviation in sympathovagal balance to sympathetic activity, might precede incident type 2 diabetes^6^.

## RELATIONSHIP BETWEEN CAN AND DEVELOPMENT OF METS

Although its prevalence is unknown, CAN can occur in MetS. Patients with diabetes have at least one component of MetS (elevated fasting glucose ≥100 mg/dL). Thus, diabetes and MetS have a common root of hyperglycemia, which is considered the most important risk factor for autonomic dysfunction. In addition, there is growing evidence that each component of MetS can contribute to development of CAN.

Recently, it was reported that delayed HRR was an independent predictor of incident MetS^7^. In contrast to incident type 2 diabetes, HRR3 was the most potent predictor of incident MetS. It is speculated that sympathetic overactivity rather than parasympathetic dysfunction is responsible for development of MetS. A 45‐b.p.m. cut‐off point of HRR3 for incident MetS was suggested, as HRR3 value ≤45 b.p.m. corresponds to a 1.3‐fold higher risk of incident MetS compared with HRR3 values >45 b.p.m.

As sympathetic withdrawal is the key mechanism for late‐stage HRR (especially HRR3), sympathetic hyperactivity could be responsible for the delayed HRR in MetS. Sympathetic hyperactivity is related to hyperinsulinemia and/or insulin resistance^8^. In addition, sympathetic abnormality is associated with each component of MetS. Thus, autonomic dysfunction represented by delayed slow phase of HRR (particularly HRR3) might precede incident MetS and be a risk factor for development of MetS.

Insulin resistance is a key mechanism in the development of MetS and could play a critical role in the delay of HRR after exercise through hyperinsulinemia and sympathetic hyperactivity. Insulin resistance has been correlated with autonomic neuropathy^9^, and factors that indicate insulin resistance (age, waist circumference, high‐sensitivity C‐reactive protein, triglyceride and homeostasis model assessment of insulin resistance) were negatively correlated with HRR3 in multiple regression analysis. Similarly, factors reflecting mitochondrial function (peak heart rate and peak oxygen uptake) that are associated with insulin resistance were positively correlated with HRR3. Collectively, delayed HRR3 could precede the development of MetS through insulin resistance.

The other potential mechanism is impaired nitric oxide function, which could affect sympathetic activity. Considering that nitric oxide suppresses sympathetic tone^10^, dysfunctions in nitric oxide metabolism associated with MetS^11^ might be related with delayed recovery from sympathetic hyperactivity. Furthermore, it is possible that resting heart rate correlates genetically with fasting plasma glucose, fasting plasma insulin, triglyceride, body mass index, waist‐to‐hip ratio and high‐density lipoprotein cholesterol, implying a genetic relationship between HRR3 and components of MetS^12^. Thus, further studies that include genetic investigations are required to explore the mechanisms of the relationship.

Although a link between delayed HRR3 and development of MetS was observed in healthy participants, further studies are warranted and should focus on the development of autonomic nervous system dysfunction in various metabolic conditions, including each component of MetS.

**Figure 1:**
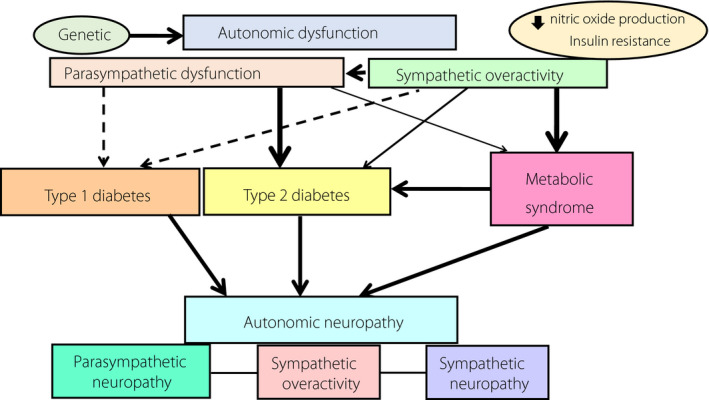
Relationships among autonomic nervous system, diabetes and metabolic syndrome. Autonomic neuropathy is a well‐known chronic complication of diabetes mellitus and might precede the development of either type 2 diabetes or metabolic syndrome. Genetic and acquired etiologies are associated with autonomic dysfunction, and either parasympathetic dysfunction or sympathetic hyperactivity could potentially lead to type 2 diabetes through a decrease in insulin secretory capacity or to metabolic syndrome through an increase in insulin resistance. Arrows show the degree of the associations, and the thicker the arrow, the stronger the evidence of association. Broken arrows denote the weakest associations.

The present editorial focused on some of the clinical studies on the associations among autonomic nervous system, type 2 diabetes and MetS, as summarized in Figure 1.

## DISCLOSURE

The authors declare no conflict of interest.

Approval of the research protocol: N/A.

Informed consent: N/A.

Approval date of registry and the registration no. of the study/trial: N/A.

Animal studies: N/A.
